# High-dose antioxidants for central serous chorioretinopathy; The randomized placebo-controlled study

**DOI:** 10.1186/1471-2415-12-20

**Published:** 2012-07-16

**Authors:** Mansing Ratanasukon, Patama Bhurayanontachai, Pichai Jirarattanasopa

**Affiliations:** 1 Department of Ophthalmology, Faculty of Medicine, Prince of Songkla University, Hat Yai, Songkhla province, 90110, Thailand

**Keywords:** Antioxidants, Central serous chorioretinopathy

## Abstract

**Background:**

To determine the efficacy of high-dose antioxidants in the acute stage of central serous chorioretinopathy (CSC).

**Methods:**

This was a randomized placebo-controlled study. The patients with acute CSC (onset within 6 weeks) were randomized to receive either high-dose antioxidant tablets (study group A) or placebo tablets (control group B) for 3 months or until the complete resolution of subretinal fluid. After 3 months, additional treatment with laser or photodynamic therapy (PDT) was considered if any fluorescein leakage persisted. The outcomes measured were the changes in visual acuity (VA) and central macular thickness (CMT), the number of patients with subretinal fluid at each follow-up time, the number of patients with fluorescein leakage at the end of the 3^rd^ month and patients who received additional treatments.

**Results:**

Fifty-one of 58 patients (88%) completed the follow-up criteria. The baseline demographic data were comparable in both groups. At the end of the 3^rd^ month, the VA and CMT showed no statistical difference between the groups but the patients in group A has less fluorescein leakage and additional treatments than in group B (p = 0.027 and 0.03).

**Conclusion:**

The high-dose antioxidants for acute CSC did not show any benefits in VA and CMT. However, the drugs might decrease the chance for fluorescein leakage and additional treatments at the end of the 3^rd^ month.

## Background

Central serous chorioretinopathy (CSC) is a serous neurosensory detachment that usually involves the macular area. It is common in patients between the ages of 30–50 years and affects males more often than females with a ratio of 2:1 [1,2]. The common risk factors are psychological stress, type A personality, systemic steroid use, hypertension and pregnancy [3-5]. The treatment is usually observation especially in the first three months. Laser or photodynamic therapy (PDT) should be considered when the condition does not improve after that time. Nevertheless, the pathogenesis of CSC is still not well understood but the study from indocyanine green (ICG) angiography showed choroidal vascular hyperpermeability and abnormal leakage [6-8]. The causes of this abnormality are supposed to come from nitric oxide, prostaglandins or even free oxidative radicals [3,6-8]. From this hypothesis, the oxidative process might be involved in the pathogenesis of the disease especially in the early stage. This study is to determine the effect of antioxidant drugs in the acute stage of CSC and to determine whether they can improve the outcome of the disease.

## Methods

**Figure 1:**
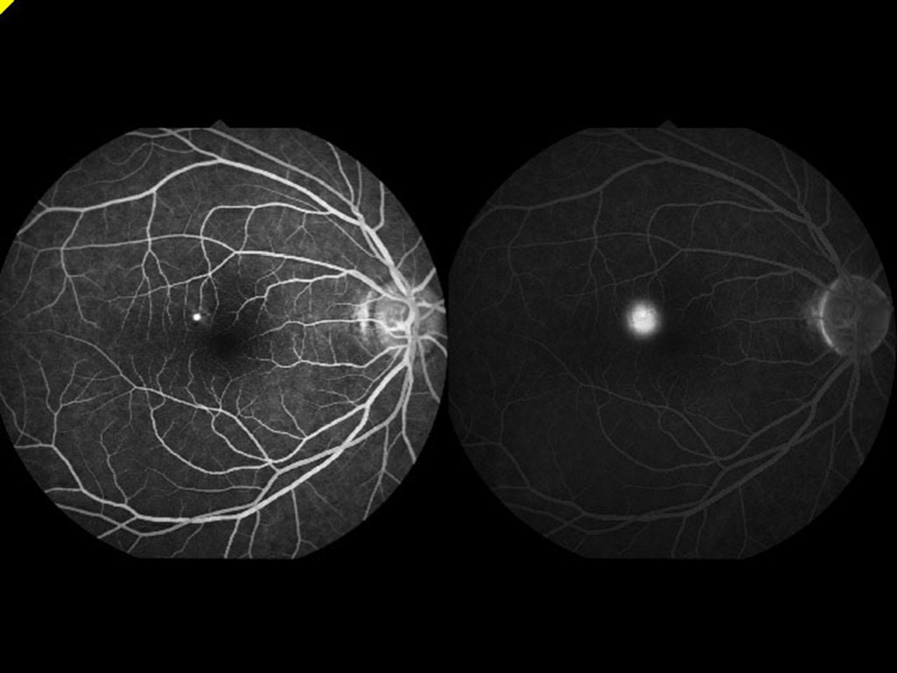
The patient with central serous chorioretinopathy showed typical inkblot leakage in the early phase (left) and late phase (right) from fluorescein angiography.

The study is a prospective, randomized, placebo-controlled study for high-dose oral antioxidants for patients who presented with acute CSC between December 2004 and December 2008 at the Retina Unit, Prince of Songkla University, Thailand. The research was approved by the Ethics Committee, Faculty of Medicine, Prince of Songkla University in December 2004 (EC 47/362-023), and before the enrollment all patients signed the consent forms. The patients were randomly assigned to high-dose antioxidant or placebo drugs. The randomization was computer generated with a 1:1 ratio, block lengths of 4 and random numbers were coded to all bottles. Moreover, the codes were in envelops until the end of the study. The inclusion criteria were patients with acute CSC (within 6 weeks of onset), aged between 30–50 years, new or recurrent attack (symptom-free 6 months or longer), a fluorescein angiography (FA) confirmed diagnosis with inkblot (Figure 1) or smokestack leakage, an optical coherence tomography (OCT) by Stratus OCT™ (Carl Zeiss Meditec, Inc., Dublin, CA) showing definite subretinal fluid and the patients’ ability for proper follow-up. The exclusion criteria were chronic CSC (longer than 6 weeks), multiple attacks (more than 2 times), large pigment epithelial detachment (more than 1 disc diameter), multiple pigment epithelial detachment or diffuse retinal pigment epitheliopathy, younger or older ages, a follow-up time less than 3 months, a complicated CSC such as secondary choroidal neovascularization detected from FA, pregnancy, steroid use and patients with contraindication for high-dose antioxidants therapy such as heavy smokers, those with lung cancer, thyrotoxicosis, renal stones and anemia (hematocrit less than 30%).

The study group (group A) received high-dose antioxidant tablets (Icaps^TM^, Alcon Laboratories) that contained vitamin A (6600 IU), vitamin C (400 mg), vitamin E (150 IU), riboflavin (10 mg), zinc (60 mg), copper (4 mg), selenium (40 mg), manganese (4 mg) and lutein/zeaxanthin (4000 micrograms). The drug was taken immediately after diagnosis, 1 tablet two times a day after meals for 3 months or until the complete resolution of subretinal fluid as assessed by OCT.

The control group (group B) received placebo tablets that were specially made for the study and were identical to the study tablets in their size, color and identification name and were taken in similar ways as the study drugs. Both drugs were also in identical opaque bottles and prepared by one non-clinician research assistant.

The study was an intention-to-treat analysis and there was no cross-over of patients between the groups after the study. The corresponding author (M.R.) generated the allocation sequence, enrolled the patients and assigned patients to any additional treatments when needed.

**Figure 2:**
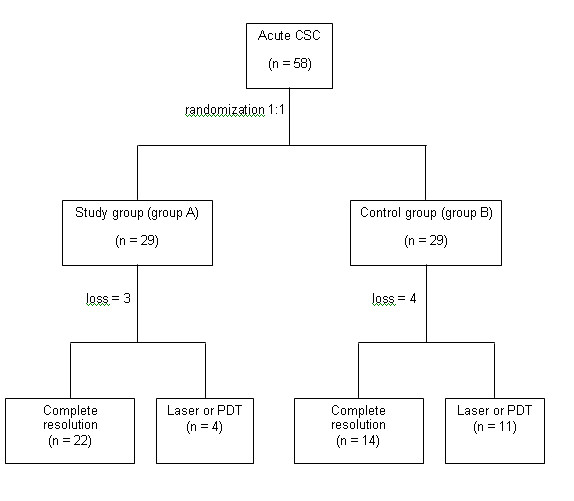
**The trial profile CSC = central serous chorioretinopathy,PDT = photodynamic therapy Study group (group A): the group received antioxidant tabletsControl group (group B): the group received placebo tablets.**

The patients were scheduled for follow-up every month. The visual acuity (VA), using the logarithm of minimum angle of resolution (logMAR), OCT using central macular thickness (CMT), FA and any complications were assessed during each visit. The study drug or placebo drug was discontinued in a patient when OCT demonstrated complete resolution of subretinal fluid or at the end of the 3^rd^ month after finishing 3 bottles of the drug. After 3 months, additional treatments such as laser photocoagulation for extrafoveal leakage or PDT for juxtafoveal or subfoveal leakage were considered for any persistent FA leakage (Figure 2).

The primary outcomes were the changes in VA and CMT recorded by OCT during every visit. The secondary outcomes were the number of patients with subretinal fluid at each follow-up time, the number of patients who showed FA leakage at the end of the 3^rd^ month and patients who received additional treatments in each group.

The sample size was calculated with α = 0.05 (two-sided), β = 0.2 and determined to be approximately 24 patients in each group. Assuming an expected dropout rate of 20%, 29 patients in each group were needed. An independent *t*-test was used for parametric data. The chi-square and Mann Whitney *U*-test were used for non-parametric data. A p-value less than 0.05 was considered significant.

## Results

A total of 58 eyes in 58 patients were included in the study and were randomized equally with 29 eyes in each group. Seven eyes (12%) were lost to follow-up or did not have enough data for analysis at the 3^rd^ month (3 eyes in the study group and 4 eyes in the control group). A total of 51 eyes (88%) were included in the final analysis (Figure 2).

The mean age and duration of CSC before enrollment of both groups were 40.4 years and 15.4 days. Although the study group showed a lower mean onset duration time than the control group (13.2 vs 17.5 days), the p-value demonstrated no statistical difference (p = 0.09). The mean baseline VA, CMT as determined by OCT and follow-up time in both groups were 0.25, 516 microns and 8.6 months, respectively. Most of OCT findings in both groups showed subretinal fluid and/or intraretinal edema but very few cases had cystoid macular edema. The baseline fluorescein leakage pattern of most cases (52 eyes, 90%) was inkblot leakage and only 6 eyes (10%, 3 in each group) demonstrated a smokestack pattern. The baseline demographic data are summarized in Table 1 and show no statistical significance between any of the parameters for both groups.

**Table 1:** Demographic Data of the Study and Control Groups

	**Study group (group A)**	**Control group (group B)**	**p-value**
Age (years ± SD)	41.28 ± 5.07	39.48 ± 6.95	0.27
Sex (M/F)	23/6	25/4	0.49
Duration before diagnosis (days ± SD)	13.24 ± 9.65	17.55 ± 9.66	0.09
Initial VA (logMAR ± SD)	0.23 ± 0.27	0.27 ± 0.20	0.59
Initial CMT (microns ± SD)	524.28 ± 175.95	508.69 ± 135.31	0.70
FA findings at baseline			
- inkblot leakage	26	26	-
- smokestack leakage	3	3	1.00

Study group (group A): the group received antioxidant tablets.

Control group (group B): the group received placebo tablets.

SD = standard deviation.

M = male, F = female, VA = visual acuity.

logMAR = logarithm of minimum angle of resolution.

CMT = central macular thickness.

FA = fluorescein angiography.

**Figure 3:**
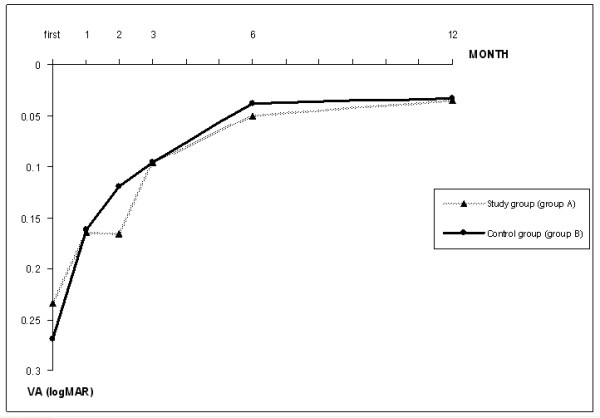
The mean visual acuity (VA) at each follow-up time between the study and control group. Study group (group A): the group received antioxidant tablets, Control group (group B): the group received placebo tablets, VA = visual acuity, logMAR = logarithm of minimum angle of resolution.

**Figure 4:**
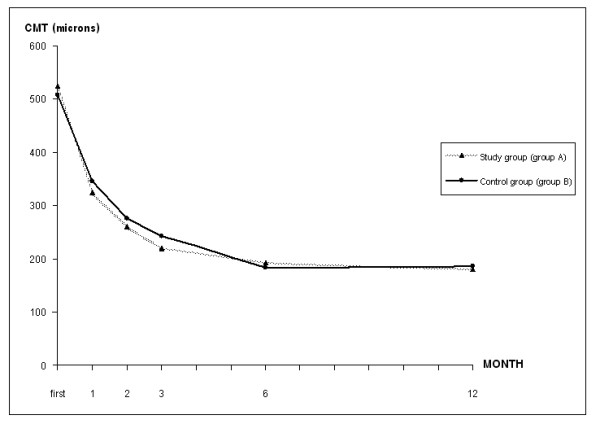
The mean central macular thickness (CMT) at each follow-up time between the study and control group. Study group (group A): the group received antioxidant tablets, Control group (group B): the group received placebo tablets, CMT = central macular thickness.

At the end of the 3^rd^ month, all patients showed no significant side effects. The VA and CMT in both groups also showed no statistical differences (p = 1.0 and 0.25, Figure 3, 4). Regarding the secondary outcomes, the number of eyes with subretinal fluid at the 3^rd^ month was higher in the control group than the study group (Table 2). Moreover, the number of eyes that demonstrated fluorescein leakage at the end of the 3^rd^ month was also higher in the control group (10 eyes) than in the study group (3 eyes) (odds ratio, 0.19; 95% CI, 0.046-0.83; p = 0.027) (Table 2). These patients were the same group of the patients with subretinal fluid at the 3^rd^ month. These results imply that the study drug might affect the leakage activity more than the fluid (both intraretinal and subretinal) absorption by retinal pigment epithelium. With regards to the recurrent rate, 3 patients developed a recurrent attack: 1 eye in the study group and 2 eyes in the control group (p = 0.29). Two of them (one in each group) had received additional treatments at the end of the 5^th^ and 8^th^ month, whereas another one had spontaneous resolution of subretinal fluid without any treatment.

**Table 2:** The Study Results

	**Study group (group A)**	**Control group (group B)**	**p-value**	**Odds ratio**	**95% CI**
Number of patients -at 1^st^ month	26	29	0.2	-	-
-at 3^rd^ month	26	25	1.00		
-at 6^th^ month	16	14	0.79		
-at 12^th^ month	7	7	1.00		
VA (logMAR ± SD) -at 3^rd^ m.	0.09 ± 0.20	0.09 ± 0.09	0.99	-	-
-at 6^th^ m.	0.05 ± 0.06	0.04 ± 0.06	0.60		
-at 12^th^ m.	0.04 ± 0.05	0.03 ± 0.05	0.91		
CMT (microns ± SD) -at 3^rd^ m.	220.65 ± 94.37	243.84 ± 76.13	0.34	-	-
-at 6^th^ m.	193.20 ± 46.38	183.22 ± 22.59	0.41		
-at 12^th^ m.	180.00 ± 15.05	186.22 ± 20.62	0.42		
Patients with subretinal fluid (number of patients)					
-at 1^st^ month	22	23	0.73	-	-
-at 2^nd^ month	13	14	0.78	-	-
-at 3^rd^ month	3	10	0.027	0.196	0.046-0.830
Follow-up time (m ± SD)	9.04 ± 6.69	8.14 ± 8.69	0.67	-	-
FA leakage at 3^rd^ m. (patients)	3	10	0.027	0.196	0.046 - 0.830
- inkblot leakage	2	9	-	-	-
- smokestack leakage	1	1	0.94	-	-
Laser or PDT (number of patients)	4	11	0.03	0.231	0.061 - 0.872
Recurrence (number of patients)	1	2	0.29	-	-

Study group (group A): the group received antioxidant tablets.

Control group (group B): the group received placebo tablets.

CI = confidence interval.

VA = visual acuity, logMAR = logarithm of minimum angle of resolution.

SD = standard deviation.

m = month, CMT = central macular thickness.

FA = fluorescein angiography, PDT = photodynamic therapy.

Because of the difference in leakage activities after 3 months of randomization, 4 patients in the study group and 11 patients in the control group were considered for additional laser treatment or PDT (including 2 recurrent cases). The p-value also demonstrated significance (odds ratio, 0.23; 95% CI, 0.061-0.872; p = 0.03) in terms of these additional treatments (Table 2). Thereafter, although the study showed no significance between the final VA and CMT as determined by OCT, the results showed that the patients who received high-dose antioxidants in the acute phase of the disease had less of a chance of having subretinal fluid and being treated by laser or PDT at the end of the 3^rd^ month.

## Discussion

Central serous chorioretinopathy (CSC) is a disease that affects young patients and sometimes results in chronic maculopathy that might permanently affect visual acuity. The disease usually resolves itself within three months after the onset and treatment using laser or PDT should be considered only after that time [1,9,10]. Before the end of the 3^rd^ month, there are many modalities studied to treat CSC such as multivitamins and acetazolamide tablets. Pikkle et al. demonstrated the effect of acetazolamide in treating CSC and showed some benefit when compared with placebo [11]. Moreover, there is some evidence that the oxidative process might be the cause of RPE/choroidal abnormality and subretinal fluid in CSC [3,6-8], so the main objective in this study is to postulate a new drug modality in treating acute onset CSC and to determine whether the drug can improve the visual outcomes and/or decrease the chance of laser treatment or PDT at the end of the 3^rd^ month.

Various mechanisms or processes have been proposed for the pathogenesis of CSC. A study from indocyanine green (ICG) angiography demonstrated that choroidal abnormalities such as dilatation and leakage were supposed to be the primary affected areas. These abnormalities could induce secondary RPE changes and result in the accumulation of intraretinal and subretinal fluid [6]. Laser treatment, however, was aimed to reduce subretinal fluid by direct photocoagulation to decrease any fluorescein leakage but did not affect directly the entire choroidal vessel abnormalities. PDT is the first to treat the choroidal leakage directly and gave good results [9,10]. Nevertheless, the exact mechanisms of these choroidal abnormalities are still unknown. The previous studies proposed that choroidal ischemia and/or an abnormality in the autoregulation of choroidal blood flow from nitric oxide, prostaglandins and free radicals from the oxidative process might be involved in the pathogenesis [3,6-8]. Therefore, medications such as the available antioxidant tablets might be effective in decreasing the choroidal leakage especially in the early stage of the disease.

This study was conducted only for the acute stage of CSC (less than 6 weeks of onset) with the mean time of duration of about 13.2 and 17.5 days in the study group and control group (p = 0.09). The study also excluded cases with complicated CSC such as diffuse pigment epitheliopathy or large pigment epithelial detachment. This means that the patients in both groups should have similar patterns of the disease in terms of pathogenesis, disease progression and even prognosis. At every follow-up, the VA and CMT determined by OCT of both groups showed no statistical significance. This might allow one to conclude that the antioxidant drugs do not have any beneficial effects on acute CSC. However, this conclusion should be accepted with caution because at the end of the 3^rd^ month many patients especially in the control group received additional treatments due to persistent fluorescein leakage. Thereafter, the final visual outcomes and CMT were modified by treatments such as laser or PDT at that time and might affect primary outcomes. Moreover, the CMT is the combined measurement of both intraretinal and subretinal fluid of the central macula so it might not reflect the CSC activity after the treatments, such as the rate of subretinal fluid absorption, especially at the end of the 3^rd^ month. With this in mind, we also conducted the study noting the number of patients with subretinal fluid at each follow-up time, fluorescein leakage patterns and the need for additional treatments for the secondary outcomes.

The fluorescein patterns from the study demonstrated interesting results. At the end of the 3^rd^ month, 3 patients in the study group and 10 patients in the control group still had fluorescein leakage (p = 0.027, Table 2). These results followed the difference in the number of patients with subretinal fluid at the same follow-up time and led to more chances of patients in the placebo group having additional treatments such as laser or PDT. These small group of slower-resolution might be caused by various reasons such as the difference in patients’ personality, stress, smoking, or even oxidative stress. As mentioned above, the previous studies [3,6-8] proposed the oxidative process to be involved in the condition and it might cause some patients to have slower resolution than expected. However, the oxidative process might show different effect in various structures of the retina. If the choroid were attacked by oxidative radicals, this could have secondary effects on the RPE, followed by the accumulation of both intraretinal and subretinal fluid. The oxidative process might have a greater effect in choroidal abnormalities than the RPE pumping activity. This leads patients in the control group to have a higher chance of having subretinal fluid and persistent fluorescein leakage than in the study group. It was also confirmed from the study that the final CMT by OCT that measured both intraretinal and subretinal fluid demonstrated a similar pattern of slow resolution in both groups and showed no statistical significance between the groups in any of the follow-up periods. This means that the antioxidant drugs decreased the choroidal leakage (which clinically presented as subretinal fluid and fluorescein leakage) and the resolution of intraretinal fluid that was partly affected by the RPE pumping activity was modified less by the drugs and led to the same CMT study results between the groups. Therefore, antioxidant drugs might have a more beneficial effect on choroidal leakage than RPE pumping activity and give a patient less of a chance to have persistent fluorescein leakage and additional treatments. However, as mentioned, this study did not take into account the complicated or chronic CSC so the role of antioxidant drugs in such conditions is unknown.

There are some limitations in this study. Some patients were lost from the schedule and the follow-up time was short. Although the recurrent cases were not significant between the groups (p = 0.29), the recurrent rate in the study was still inconclusive because the sample size of recurrence of only 3 eyes was too small for analysis. A long term follow-up will be organized in the near future to answer the questions of any difference in recurrent rates and any long-term side effects that might have occurred by using the drugs. Another limitation is the use of time domain OCT instead of spectral domain OCT. The previous generation of OCT machines cannot evaluate well the quantification of the subretinal fluid pockets. A future trial using spectral domain OCT would be beneficial as it would better allow evaluation of the fluid via editing segmentation. The difference in the fluorescein leakage patterns is also a limitation in this study. Because most of the cases were inkblot leakage (90%) and only 10% showed smokestack pattern we could not do a subgroup analysis of the two leakage patterns.

## Conclusions

Although the antioxidant drug treatment does not have a benefit in visual acuity and central macular thickness in acute CSC, there might be less of a chance for persistent fluorescein leakage and the need for additional treatments such as laser or PDT. A randomized controlled study with a longer follow-up and quantitative analysis of the subretinal fluid is warranted to investigate antioxidants as a possible treatment modality.

## Competing interest

The authors declare that we have no competing interests. The authors have no financial support or financial interest from any government or non-government company.

## Authors’ contributions

MR (AB, ES), PB(FG), PJ(FG). All authors read and approved the final manuscript.

## Pre-publication history

The pre-publication history for this paper can be accessed here:

http://www.biomedcentral.com/1471-2415/12/20/prepub
